# Analysis of complete mitochondrial genomes from extinct and extant rhinoceroses reveals lack of phylogenetic resolution

**DOI:** 10.1186/1471-2148-9-95

**Published:** 2009-05-11

**Authors:** Eske Willerslev, M Thomas P Gilbert, Jonas Binladen, Simon YW Ho, Paula F Campos, Aakrosh Ratan, Lynn P Tomsho, Rute R da Fonseca, Andrei Sher, Tatanya V Kuznetsova, Malgosia Nowak-Kemp, Terri L Roth, Webb Miller, Stephan C Schuster

**Affiliations:** 1 Centre for Ancient Genetics, University of Copenhagen, Universitetsparken 15, DK-2100, Denmark; 2 Centre for Macroevolution and Macroecology, School of Biology, Australian National University, Canberra, ACT 0200, Australia; 3 Pennsylvania State University, Center for Comparative Genomics and Bioinformatics, 310 Wartik Building, University Park, PA 16802, USA; 4 Department of Integrative Biology, University of California, Berkeley, CA 9472-3140, USA; 5 Severtsov Institute of Ecology and Evolution, Russian Academy of Sciences, 33 Leninsky Prospect, 119071 Moscow, Russia; 6 Department of Paleontology, Faculty of Geology, Lomonosov Moscow State University, Leninskiye Gory, 119992 Moscow, Russia; 7 Oxford University Museum of Natural History, Parks Road, Oxford, OX1 3PW, UK; 8 Center for Conservation and Research of Endangered Wildlife (CREW), Cincinnati Zoo & Botanical Garden, 3400 Vine St, Cincinnati, OH 45220, USA

## Abstract

**Background:**

The scientific literature contains many examples where DNA sequence analyses have been used to provide definitive answers to phylogenetic problems that traditional (non-DNA based) approaches alone have failed to resolve. One notable example concerns the rhinoceroses, a group for which several contradictory phylogenies were proposed on the basis of morphology, then apparently resolved using mitochondrial DNA fragments.

**Results:**

In this study we report the first complete mitochondrial genome sequences of the extinct ice-age woolly rhinoceros (*Coelodonta antiquitatis*), and the threatened Javan (*Rhinoceros sondaicus*), Sumatran (*Dicerorhinus sumatrensis*), and black (*Diceros bicornis*) rhinoceroses. In combination with the previously published mitochondrial genomes of the white (*Ceratotherium simum*) and Indian (*Rhinoceros unicornis*) rhinoceroses, this data set putatively enables reconstruction of the rhinoceros phylogeny. While the six species cluster into three strongly supported sister-pairings: (i) The black/white, (ii) the woolly/Sumatran, and (iii) the Javan/Indian, resolution of the higher-level relationships has no statistical support. The phylogenetic signal from individual genes is highly diffuse, with mixed topological support from different genes. Furthermore, the choice of outgroup (horse *vs *tapir) has considerable effect on reconstruction of the phylogeny. The lack of resolution is suggestive of a hard polytomy at the base of crown-group Rhinocerotidae, and this is supported by an investigation of the relative branch lengths.

**Conclusion:**

Satisfactory resolution of the rhinoceros phylogeny may not be achievable without additional analyses of substantial amounts of nuclear DNA. This study provides a compelling demonstration that, in spite of substantial sequence length, there are significant limitations with single-locus phylogenetics. We expect further examples of this to appear as next-generation, large-scale sequencing of complete mitochondrial genomes becomes commonplace in evolutionary studies.

"The human factor in classification is nowhere more evident than in dealing with this superfamily (Rhinocerotoidea)." G. G. Simpson (1945)

## Background

Despite being a long-standing target of scientific research, resolution of the phylogeny of the five living rhinoceroses using traditional (non-DNA) approaches has been controversial. At the root of the problem is the placement of the Sumatran rhinoceros (*Dicerorhinus sumatrensis*), a species that has retained many ancestral morphological characters, among the broadly accepted sub-clades of the black (*Diceros bicornis*) and white (*Ceratotherium simum*) rhinoceroses, and the Javan (*Rhinoceros sondaicus*) and Indian (*Rhinoceros unicornis*) rhinoceroses. For example, on the one hand, the two horns of the Sumatran rhinoceros suggest that it should be placed with the similarly two-horned black and white rhinoceroses, rather than with the single-horned Javan and Indian rhinoceroses [1,2]. On the other hand, the geographic distribution of the Sumatran rhinoceros, and its close proximity with the two other living Asian species, would indicate that they form a natural clade [3]. Third, a hard trichotomy has been proposed, reflecting an effectively simultaneous divergence of the three lineages [4-6]. Attempts to resolve such questions can be made by including fossil taxa, for example the woolly rhinoceros in this case. However, this has proven to be similarly problematic. Although it seems clear that the woolly and Sumatran are closely related (for example both have two horns and a hairy pelt), the addition of morphological information from the woolly rhinoceros has failed to produce a convincing resolution of the relationships among the three pairs.

In response to these problems, several DNA-based studies have been undertaken on the rhinoceroses in an attempt to resolve the phylogeny. The first such study used restriction-digest mapping of mitochondrial DNA (mtDNA) ribosomal region to find weak support (maximum parsimony bootstrap support of 57%) for the extant rhinoceros phylogeny as outlined on the basis of horn morphology [7]. As larger amounts of data were incorporated into the analyses, however, this picture was modified – using complete 12S rRNA and cytochrome *b *sequences, Tougard *et al. *[8] found high support (maximum likelihood bootstrap support of 97%) for the phylogeny as outlined by geography (although they could not, using a Kishino-Hasegawa test, reject outright the horn topology). More recently, Orlando *et al. *[9] analysed ancient DNA to confirm the monophyly of the woolly-Sumatran rhinoceros pairing using complete 12S rRNA and partial cytochrome *b *gene sequences (maximum likelihood bootstrap support between 93–100%). Furthermore, in agreement with the work of Tougard *et al. *[8], their inferred phylogeny groups the woolly-Sumatran pair with the Javan-Indian pair, but with <50% bootstrap support. Thus the results of these later studies appeared to be an excellent illustration of the advantages of molecular sequence analysis over more traditional approaches, when resolving subtle phylogenetic questions.

Despite the successful results, however, one of the key lessons of the above is that even when using DNA data, results can still be misleading without sufficiently large amounts of sequence. It has previously been advocated that phylogenies based on single genes can sometimes yield falsely supported results [10]. The variation in phylogenetic signal among mitochondrial genes in elephantids provides a compelling illustration of this problem [11]. In addition, Cummings *et al. *[12] analysed complete genomes and subsamples of them, concluding that small increases in sequence length will greatly increase the chance of finding the correct whole-genome tree when sequence lengths are below 3,000 base-pairs (bp). In light of this, the clearly controversial phylogeny of the Rhinocerotidae, and the fact that previous studies have maximally been based on the 2,146 bp of the combined 12S rRNA and cytochrome *b *genes, we have revisited the molecular analysis using six complete mtDNA genome sequences of the five extant, and extinct woolly, rhinoceroses. Specifically, we have generated, using our previously published approach of utilising keratinous tissues as a high-quality source of mtDNA for sequencing on the FLX platform [13-15], four novel complete mitochondrial genomes (from the black, woolly, Javan, and Sumatran rhinoceroses). With the addition of the published mtDNA genomes of the white [GenBank:Y07726] [16] and Indian [Genbank:X97336.1] [17] rhinoceroses, the six genomes cover all the living and one extinct member of the rhinocerotid family. We demonstrate that phylogenetic analysis of the complete mtDNA genomes, as well as individual analyses of the constituent genes, questions the findings of the previous molecular reports.

## Results

### Mitochondrial genome sequences

The four newly sequenced mitochondrial genomes are similar to the two previously published rhinoceros mitochondrial genomes, consisting of 13 protein-coding genes, 22 tRNA genes, two ribosomal RNA genes, and a control region. The exact length is difficult to determine due to the presence of variable tandem repeats in the control; the consecutive repeats are longer than the read length of the Roche FLX, so we are unable characterise them. The sequences have been submitted to GenBank with accession numbers FJ905813 (woolly rhinoceros), FJ905814 (black rhinoceros), FJ905815 (Javan rhinoceros) and FJ905816 (Sumatran rhinoceros).

An obvious power of complete mtDNA genome sequencing is that it enables functional assessment and comparison of the genes between the taxa [14,18]. Mitochondrial protein-coding genes are involved in oxidative phosphorylation, which is responsible for the production of up to 95% of the energy of eukaryotic cells, and modifications in these genes have been associated with the improvement of aerobic capacity and adaptation to new thermal environments [19,20]. Furthermore, mutations in mitochondrial genes have been implicated in exercise intolerance in humans [21]. We have mapped the amino acid differences between the rhinoceroses on the available crystallographic structures for mitochondrial-encoded proteins, those for *cytb *[22] and *co1*, *co2*, and *co3 *[23].

Despite some of the differences occurring in functionally relevant sites (boxed residues in the alignment, orange spheres in the structure; Figures S1 and S2: Additional Files 1 and 2), we were unable to observe any direct relationship between them and the markedly different environments inhabited by the woolly, in contrast to the extant rhinoceroses.

### Phylogeny and speciation times of the rhinoceros

The mitochondrial genomes of the six rhinoceros species were analysed using Bayesian and likelihood-based phylogenetic methods. In order to infer the position of the root, the sequences of two perissodactylan outgroup species were included in the analyses (tapir, *Tapirus terrestris*; and horse, *Equus caballus*). We find very strong support for each of the three sister-species pairings among the rhinoceroses (100% Bayesian posterior probability and maximum-likelihood bootstrap support, regardless of the choice of outgroup). This is in agreement with previous molecular findings and most morphological reports.

**Figure 1:**
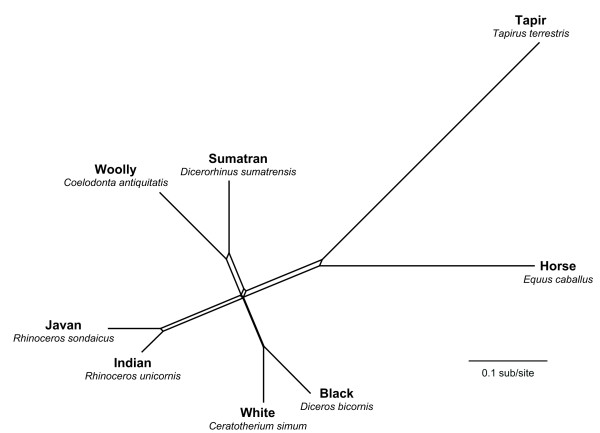
**Splits graph obtained using split decomposition analysis of whole mitochondrial genomes**. The GTR+I+G model of nucleotide substitution was assumed, using maximum-likelihood estimates of model parameters. A very similar graph was obtained using LogDet-transformed distances, but is not shown here.

Split decomposition produced a graph with good representation of the phylogenetic signal (fit value = 89.64), but with an obvious lack of resolution among the three pairs of rhinoceros species (Figure 1). Similar graphs were produced using split decomposition with other distance measures, including LogDet-transformed distances (results not shown).

Bayesian and maximum-likelihood phylogenetic analyses of the concatenated data set revealed mixed support for three candidate topologies (Table 1). The relative topological support varied according to outgroup choice, although support was generally weak. In only one case (Bayesian analysis with horse outgroup) was there significant support for a particular tree topology. Likelihood-based tree topology tests, performed using the concatenated alignment with both outgroups, yielded no significant support for any particular tree topology. There was slight (but non-significant) support in favour of the topology in which the white, black, Sumatran, and woolly rhinoceroses group together to the exclusion of the Indian and Javan rhinoceroses (approximately-unbiased test p = 0.716, Kishino-Hasegawa test p = 0.663, Shimodaira-Hasegawa test p = 0.802). There was low support for the grouping of the white, black, Indian, and Javan rhinoceroses (AU-test p = 0.398, KH-test p = 0.337, SH-test p = 0.473), and for the grouping of the Indian, Javan, Sumatran, and woolly rhinoceroses (AU-test p = 0.154, KH-test p = 0.151, SH-test p = 0.245). Regardless of the topology test used, all three candidate topologies were always included in the 95% confidence set.

**Table 1:** Support for three candidate topologies estimated using Bayesian and maximum-likelihood phylogenetic analysis

	**Bayesian posterior probability**			**Maximum-likelihood bootstrap support**		
	
**Outgroup**						
Horse	0.961	0.035	0.004	0.342	0.257	0.401
Tapir	0.050	0.469	0.480	0.074	0.171	0.755
Both	0.032	0.244	0.724	0.084	0.259	0.657
Both^a^	0.165	0.474	0.361	0.171	0.362	0.457
Both^b^	0.003	0.820	0.177	0.000	0.167	0.813
Both^c^	0.013	0.938	0.048	0.075	0.244	0.681

^a^Control region excluded

^b^All third codon sites excluded

^c^Control region and all third codon sites excluded

**Figure 2:**
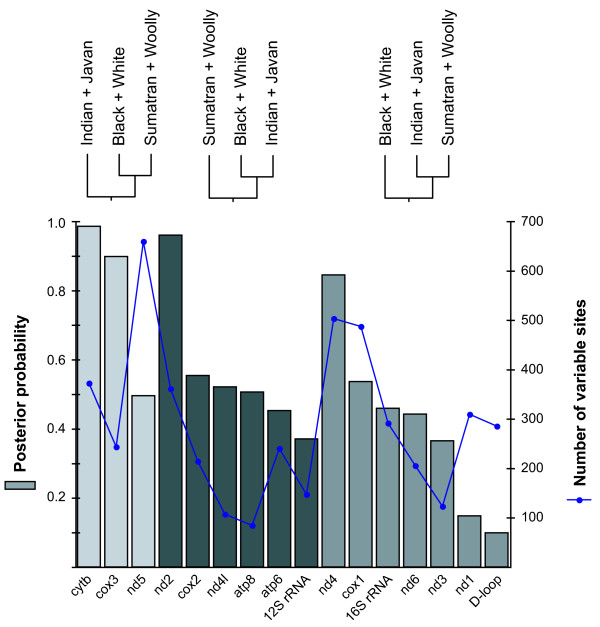
**Column graph showing posterior probability support for three candidate tree topologies**. Each column represents the posterior probability of the favoured tree for each component of the mitochondrial genome (13 protein-coding genes, loop regions of two rRNA genes, and the D-loop). The three shades of grey correspond to the three tree topologies shown above the graph. The line graph indicates the number of variable sites in each component.

**Figure 3:**
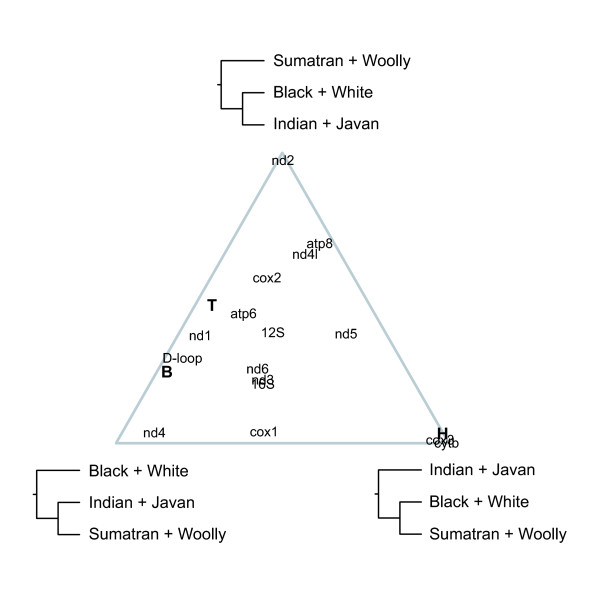
**Ternary plot showing the relative Bayesian posterior probabilities of three candidate tree topologies**. The estimated support is shown for individual components of the mitochondrial genome (13 protein-coding genes, loop regions of two rRNA genes, and the D-loop). Relative support is also shown for a concatenated data set comprising these components, with results from three analyses using different outgroup taxa (H = horse, T = tapir, B = both).

**Figure 4:**
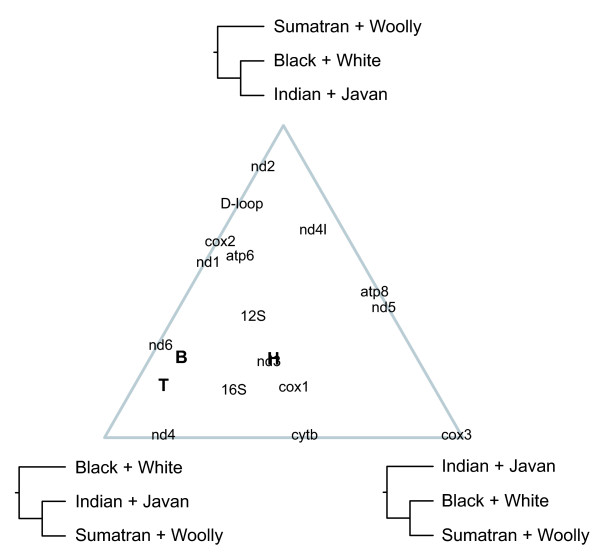
**Ternary plot showing the relative maximum-likelihood bootstrap support for three candidate tree topologies**. The estimated support is shown for individual components of the mitochondrial genome (13 protein-coding genes, loop regions of two rRNA genes, and the D-loop). Relative support is also shown for a concatenated data set comprising these components, with results from three analyses using different outgroup taxa (H = horse, T = tapir, B = both).

Analyses using individual components of the mitochondrial genome revealed wide variations in relative support for the three candidate topologies (Figures 2, 3, 4). Several genes exhibited strong support for particular topologies: *nd2*, *cox3*, and *cytb *in the Bayesian analyses, and *cox3 *in the maximum-likelihood analyses.

Bayesian estimates of divergence times within Rhinocerotidae, calculated using two fossil-based calibrations, were similar across different tree topologies (Table 2). The estimated age of the common ancestor of the six rhinoceros taxa was around 30 Myr, with a 95% highest posterior density (HPD) interval of about 28–33 Myr. The next divergence within the group occurred only around 1.0–1.5 Myr afterwards, with a very considerable overlap in the 95% HPD intervals of these two adjacent nodes (nodes A and B in Table 2). On average across the three topologies, the mean time separating these two nodes represents only 3.78% of the total tree height.

**Table 2:** Rhinocerotid divergence times estimated using Bayesian phylogenetic analysis with a relaxed molecular clock, assuming each of three candidate tree topologies

	Date estimate (mean and 95% HPD*)					
	
**Divergence event**						
Indian-Javan	13.2	(11.7 – 14.6)	13.4	(11.7 – 15.2)	12.9	(11.5 – 14.2)
Black-White	15.3	(13.5 – 17.4)	15.9	(13.5 – 18.5)	15.1	(13.6 – 16.7)
Woolly-Sumatran	19.8	(17.8 – 21.9)	20.6	(18.4 – 22.8)	19.6	(17.5 – 21.5)
Node A	30.6	(28.0 – 33.3)	30.5	(27.7 – 33.4)	30.4	(28.0 – 33.0)
Node B	31.6	(29.2 – 34.3)	32.0	(29.1 – 34.8)	31.5	(29.0 – 34.0)

*95% highest posterior density interval

Time is in millions of years before the present.

## Discussion

As ancient DNA techniques have advanced, the complete mitochondrial genomes of extinct taxa are now regularly being included in DNA analyses. Initial complete ancient mtDNA genomes were generated using conventional overlapping-PCR and Sanger sequencing techniques, yielding the mtDNA genomes of three moa species (*Emeus crassus, Anomalopteryx didiformis*, and *Dinornis giganticus*) [24,25], several woolly mammoths (*Mammuthus primigenius*) [26,27], and the mastodon (*Mammut americanum*) [11]. These genomes were successfully used to resolve long-standing phylogenetic questions, including the ratite and Elephantidae phylogenies. Notably, both questions were those for which prior analysis of smaller mitochondrial and nuclear fragments had yielded contradicting results [28-33]. In light of these successes, therefore, our inconclusive findings come as a surprise. Previous morphological studies, along with restriction enzyme mapping studies, have supported the horn-based topology grouping the black and white rhinoceroses with the Sumatran and woolly rhinoceroses [1,2,7]. More recent studies of morphological and DNA data have favoured the geography-based topology, grouping all the Asian species together to the exclusion of the African species [3,8]. With the complete mitochondrial genomes, however, the third possible candidate grouping (the African species together with the Indian and Javan rhinoceroses) cannot be statistically rejected.

The discrepancy between the results of the present and previous studies led us to investigate the topologies supported by each individual mitochondrial gene. The signal varied considerably among genes, with 3–7 genes supporting each of the three candidate topologies (Figures 1, 2, 3, 4). The considerable heterogeneity in topological support among mitochondrial genes is unusual due to the non-recombining mode of genomic transmission, but echoes similar results obtained for elephantids [11] and for chloroplast genes in *Asplenium *ferns [34]. A possible explanation for the lack of resolution could be the short divergence time among the three sister-species pairings of only ~1 Myr, leaving little time to accumulate mutations in the ancestral branches; thus, the poor resolution might be indicative of a hard polytomy. The cause of the multiple defining speciation events of the rhinoceros family is unknown, but can be identified as having occurred during the early Oligocene (33.9 ± 0.1 – 28.4 ± 0.1 Myr ago). A less likely explanation for the lack of phylogenetic resolution, posited by Shepherd *et al*. [34] to explain the phylogenetic conflicts among chloroplast genes in ferns, is that recombination might have occurred in the rhinocerotid mitochondrial genome. Finally, it is possible that the mitochondrial tree does not provide an accurate reflection of the underlying species phylogeny, a question that will need to be addressed using data from multiple nuclear loci.

## Conclusion

We have demonstrated that, in addition to hair, nail is an excellent substrate with which complete mitochondrial genomes can be generated from old or ancient material. Using these substrates we have generated the sequences of four previously unpublished rhinoceros mtDNA genomes. Although several previous studies of partial mtDNA genomic sequences apparently resolved the long-standing question as to the true phylogeny of the recent rhinoceros species, we have demonstrated that these estimates were probably misled by sampling error. Furthermore, even when using complete mitochondrial sequences, we are unable to resolve the tree topology. Thus, for the rhinoceroses at least, DNA analyses have not yet been able to provide a solution to the evolutionary history of this challenging taxon. Several possible steps might be taken to resolve this problem. One would be the inclusion of DNA sequences recovered from a more closely related outgroup species, such as members of the *Elasmotherium *genus. Unfortunately however, the age of known specimens likely precludes the survival of DNA in them. Thus it is probable that satisfactory resolution of the phylogeny will instead require the incorporation of substantial amounts of nuclear DNA data.

## Methods

### Samples

In our previous studies [13-15,35,36], we have reported the benefit of using hair shaft as a source of enriched, pure mtDNA, from which complete mitochondrial genomes can be rapidly sequenced using the Roche FLX technology. We have previously argued that the benefits derive principally from the keratin structure of the material. In this study we have used hair shaft, and have explored the properties of a similarly keratinous tissue, nail. Specifically, ancient permafrost-preserved hair shaft was used for the woolly rhinoceros, while modern hair shaft was donated by Cincinnatti Zoo (USA) for the Sumatran rhinoceros. The woolly rhinoceros hair was excavated from the permafrost near the Taimylyr village, not far from the Arctic Ocean coast (Yakutia, Olenyok River valley, 72.61°N and 121.93°E) in 2002 and is kept in the Lena Delta Reserve in Tiksi; the age of this specimen is not known. For the black and Javan rhinoceroses (the latter not found in captivity) we used historic museum toenail samples, dated to approximately 100 years old, and provided by the Zoological Museum, University of Copenhagen (Denmark), and the Oxford University Museum of Natural History (England), respectively. In these cases, powdered nail was obtained by drilling directly into the nails. Full sample details are given in Table 3.

**Table 3:** Details of the samples and sequencing efforts

**Species**	**Sample ID**	**Material**	**Weight (g)**	**Age (years)**	**Bases sequenced**	**%mtDNA**	**Length**	**Coverage**	**Genbank**
Black	#36	Nail	0.56	ca. 100	359,644	1.21	119.8	21.3	FJ905814
Woolly	COEL LDR P75	Hair	1.5	Not dated	498,748	6.6	91.5	30.8	FJ905813
Sumatran	Suci*	Hair	0.2	Fresh	199,235	1.14	88.2	12.1	FJ905816
Javan	4139	Nail	0.86	ca. 100	85,223	0.41	100.1	5.1	FJ905815

*Zoo studbook number 43.

### Extractions and sequencing

Prior to DNA extraction, hair shaft was thoroughly cleansed in 10% commercial bleach solution following Gilbert *et al. *[13]. The powdered nail was likewise incubated in 10% commercial bleach solution for 5 minutes, prior to pelleting through a centrifugation step. Subsequently the bleach was poured off, and the pellet was thoroughly washed three times in ddH_2_0 to remove all traces of the bleach. Both hair and nail material were digested and DNA was subsequently purified following Gilbert *et al. *[13]. Specifically, digestion of the hair shafts was performed overnight at 55°C with rotation, using between 10 and 40 ml of the following digestion buffer: 10 mM Tris-HCl (pH 8.0), 10 mM NaCl_2_, 2% w/v Sodium Dodecyl Sulfate (SDS), 5 mM CaCl_2_, 2.5 mM Ethylene-Diamine-Tetra-Acetic acid (EDTA), pH 8.0), 40 mM Dithiothreitol (DTT; Cleland's reagent) and 10% proteinase K solution (>600 mAU/ml, Qiagen). Post digestion DNA was purified twice with phenol and once with chloroform following standard protocols. The aqueous, DNA-containing solution was concentrated to 200 μl using Amicon Ultra-15 Centrifugal Filter Units with a 10 kD filter (Millipore).

### Library construction, DNA sequencing, and assembly

The DNA libraries from four rhinoceros species were constructed as previously described [37], by shearing genomic DNA into fragments which were blunt-ended and phosphorylated by enzymatic polishing using T4 DNA polymerase, T4 polynucleotide kinase, and *E. coli *DNA polymerase. The polished DNA fragments were then subjected to adapter ligation, followed by isolation of the single-stranded template DNA (sstDNA). The quality and quantity of the sstDNA library was assessed using the Agilent 2100 Bioanalyzer (Agilent Technologies, Santa Clara, CA). Each sstDNA library fragment was captured onto a single DNA capture bead, and was clonally amplified within individual emulsion droplets. The emulsions were disrupted using isopropanol, the beads without an amplified sstDNA fragment were removed, and the beads with an amplified sstDNA fragment were recovered for sequencing. The recovered sstDNA beads were packed onto a 70× 75 mm PicoTiterPlate™ and loaded onto the Roche FLX Sequencing System (Roche Applied-Sciences, Indianapolis, IN) as previously described. In total, 70,082 reads were generated for woolly rhinoceros, 565,065 for Sumatran rhinoceros, 251,087 for black rhinoceros, and 96,900 for Javan rhinoceros. Sequencing reads from each project were aligned using the complete mitochondrial genome from the white rhinoceros as a reference [GenBank:Y07726].

The DNA libraries from the four new samples yielded between 0.41 and 6.6% mtDNA sequences (Table 3), as identified through alignment with the white rhinoceros mitochondrial genome. The remaining sequence composition was not analysed in detail, due to difficulties associated with a lack of closely related nuclear genome against which to compare the results. The data indicate that, as a source of ancient mtDNA, nail is comparable to hair. Furthermore, the mtDNA yield of the woolly rhinoceros sample is the highest observed from any hair shaft studied to date.

The mtDNA genomes of the taxa were assembled using the white rhinoceros mtDNA genome as a guide, analogous to the method of Gilbert *et al. *[13]. A frequently reported problem in conventional (i.e., PCR-based) ancient mtDNA studies (in particular for the woolly rhinoceros [9]) is the PCR amplification of numts, and their subsequent erroneous designation as true mtDNA sequences. This is not, however, a problem with FLX generated sequences for reasons argued previously [13], that principally relate to the differences between the shot-gun nature of FLX emPCR and sequencing and the targeted nature of PCR based amplification. Thus we are confident the data represents the true mtDNA sequence. The genomes could be assembled as single contigs for the woolly, Javan, and black rhinoceroses, while that of the Javan rhinoceros, which yielded the lowest levels of mtDNA in this data set, was initially assembled into 15 contigs. Traditional PCR and Sanger sequencing was thus applied to the extract, both to ensure the sequence accuracy of the regions that had low levels of FLX coverage (<3×), and to fill in the missing data (Table S1: Additional File 3).

### Sequence alignment and partitioning

The mitochondrial genomes of the six rhinoceroses were aligned manually. The variable number tandem repeats (VNTRs) in the D-loop were removed, leaving a data set of 16,323 aligned sites. A matrix containing uncorrected pairwise distances for the six mitochondrial genomes is provided in Table S2 (Additional File 4), with details of informative sites given in Table S3 (Additional File 5).

To allow the position of the root to be inferred, mitochondrial genome sequences from the horse (*Equus caballus *[GenBank:X79547] [38]) and lowland tapir (*Tapirus terrestris *[GenBank:AJ428947], unpublished data deposition in Genbank [39]), were added to the alignment. The resulting data set includes representatives from all three families of Order Perissodactyla: Rhinocerotidae, Tapiridae, and Equidae. Analyses including additional representatives of Laurasiatheria were performed, without yielding qualitatively different results, are not presented here but are summarised in Table S4 (Additional File 6).

We constructed a concatenated data set (13,714 bp), with the following five partitions: (i) first codon sites of the 13 protein-coding genes; (ii) second codon sites of protein-coding genes; (iii) third codon sites of protein-coding genes; (iv) loop regions of *12S *and *16S *rRNA genes; and (v) D-loop. The RNA loop regions were defined in accordance with the RNA secondary structural models of *Ceratotherium simum *available from the European Ribosomal RNA Database [40]. The D-loop was defined using the GenBank annotation of *Ceratotherium simum*. Other sections of the mitochondrial genomes, including the RNA stems, tRNA genes, and intergenic sites were not used for further study, except in the split decomposition analysis.

### Split decomposition

To examine the overall phylogenetic signal, we analysed the alignment of complete mitochondrial genomes (excluding the VNTRs) using the software SplitsTree 4 [41]. Pairwise distances were estimated with the GTR+I+G model of nucleotide substitution, using maximum-likelihood estimates of model parameters. Using the split decomposition method [42], the data were canonically decomposed into a sum of weakly compatible splits and represented in the form of a splits graph.

### Phylogenetic analysis

We performed a Bayesian phylogenetic analysis of the concatenated sequence alignment using *MrBayes *3.1 [43]. A separate substitution model was assumed for each of the five data partitions, with substitution models selected by comparison of Bayesian information criterion scores (Table S3). Posterior distributions of parameters, including the tree topology, were estimated using Markov chain Monte Carlo (MCMC) sampling with two chains (one heated). Samples from the posterior were drawn every 1,000 MCMC steps over a total of 11,000,000 steps, with the first 1,000 samples discarded as burn-in. Acceptable mixing and convergence to the stationary distribution were checked using *Tracer *1.4 [44].

To examine the effect of outgroup choice, we ran two further phylogenetic analyses, excluding the horse and tapir sequences in turn. All other settings were identical to those of the original analysis.

Maximum-likelihood phylogenetic analysis was also performed on the concatenated data sets, using the software PAUP* 4b10 [45]. As with the Bayesian analyses, different combinations of outgroup taxa were tested (horse, tapir, and both). The GTR+I+G model of nucleotide substitution was assumed, with six rate categories for the discrete gamma distribution. Each data partition was allowed to have a unique substitution rate. In each analysis, the maximum-likelihood tree was identified using a branch-and-bound search. To assess levels of support for different nodes in the tree, bootstrap analysis was conducted with 1,000 pseudoreplicates.

Topology tests were performed in a maximum-likelihood framework using the program *Consel *[46]. This program implements the approximately-unbiased test [47], the Kishino-Hasegawa test [48], and the Shimodaira-Hasegawa test [49], among others. We used these to assess the relative confidence in three competing topological hypotheses (see Table 2). Site-wise log likelihoods were calculated using *PAUP*, with the same settings as for the analysis of the concatenated data described above.

To investigate variations in phylogenetic signal among different genes, Bayesian and maximum-likelihood phylogenetic analyses were performed for each protein-coding gene, rRNA gene (loops only), and the D-loop. Both outgroup taxa (horse and tapir) were included. Substitution models were chosen by comparison of Bayesian information criterion scores (Table S3). The same settings were used as for the analyses of the concatenated data set.

### Divergence time estimation

Rhinocerotid divergence times were estimated by Bayesian phylogenetic analysis of the concatenated sequence alignment, using both horse and tapir as the outgroup. The alignment was analysed using an uncorrelated lognormal relaxed-clock model in *BEAST *1.4.7 [50,51]. As with the *MrBayes *analysis, a separate substitution model was assumed for each of the five data partitions, with substitution models selected by comparison of Bayesian information criterion scores (Table S3). The tree topology was fixed, with a separate analysis performed for each of the three candidate trees (see Table 2). Posterior distributions of parameters were estimated using Markov chain Monte Carlo (MCMC) sampling. Samples from the posterior were drawn every 1,000 MCMC steps over a total of 5,500,000 steps, with the first 500 samples discarded as burn-in. Acceptable mixing and convergence to the stationary distribution were checked using *Tracer*.

In order to place a geological time-scale on the phylogenetic tree, two calibrations were taken from the fossil record. First, a lognormal prior (minimum 56 Myr, mean 60 Myr, standard deviation 1.51 Myr) was specified for the age of the root [52,53]. This is probably the most appropriate summarisation of paleontological information because the probability density of the nodal age has a mode that is older than the age of the oldest fossil belonging to either of the lineages descending from the node [54]. Second, a minimum age constraint of 16 Myr was placed on the *Dicerorhinus *lineage [55].

## Authors' contributions

EW, MTPG and SCS conceived the study. MTPG, PFC, LPT and SCS carried out the molecular genetic studies. MTPG, SYWH, AR, RRdF, WM and SCS carried out data analysis. AS, TVK, MN-K and TLR coordinated sampling of biological material. JB, AS, TVK, MN-K and TLR interpreted the results in the biological context. All authors wrote, read and approved the final manuscript.

## Supplementary Material

Additional file 1 **Figure S1**. Nonsynonymous sites in mitochondrial *cytb *sequences of rhinoceroses, mapped onto the bovine structure [PDF:1PPJ] [22]. The nonsynonymous sites are shown in white, with those located in functionally relevant areas represented as orange spheres, and surrounded by boxes in the alignment. The prosthetic groups are represented in black, and bound inhibitors in brown (ant: antimycin; stig: stigmatellin). Sites that, when mutated in humans, are responsible for exercise intolerance, are depicted in red. Click here for file

Additional file 2 **Figure S2**. Nonsynonymous sites in mitochondrial *co1*/*co2*/*co3 *sequences of rhinoceroses, mapped onto the bovine structure [PDF:1V54] [23]. The nonsynonymous sites are shown in white, with that located in a functionally relevant area represented as orange spheres, and surrounded by a box in the alignment. Prosthetic groups are represented as grey spheres. Click here for file

Additional file 3 **Table S1**. Details of the primers used for gapfilling of the Javan rhinoceros mitochondrial genome. Click here for file

Additional file 4 **Table S2**. Matrix of uncorrected p-distances for the whole mitochondrial genomes of six rhinoceros species, with distances between sister species given in bold. Click here for file

Additional file 5 **Table S3**. Details of the components of the aligned mitochondrial genomes from six rhinoceroses, tapir, and horse. Click here for file

Additional file 6 **Table S4**. Support for three candidate topologies estimated using Bayesian and maximum-likelihood phylogenetic analysis, using nine laurasiatherian outgroup species. In addition to the six rhinoceros sequences, the following outgroup taxa were included: *Tapirus terrestris *[GenBank:AJ428947], *Equus caballus *[GenBank:X79547], *Hippopotamus amphibius *[GenBank:NC_000889], *Equus asinus *[GenBank:NC_001788], *Artibeus jamaicensis *[GenBank:NC_002009], *Ursus arctos *[GenBank:NC_003427], *Manis tetradactyla *[GenBank:NC_004027], *Bos taurus *[GenBank:NC_006853], and *Sorex unguiculatus *[GenBank:NC_005435]. Phylogenetic analyses were performed on first and second codon positions only, using an unpartitioned GTR+I+G substitution model. Other settings in the Bayesian and likelihood-based analyses were as described in the main text, the only exceptions being the use of the heuristic tree-bisection-reconnection instead of a branch-and-bound search in the maximum-likelihood analysis, and the calculation of the bootstrap support values from 200 pseudoreplicates rather than 1,000. Click here for file
