# Supplementary material for: Analysis of complete mitochondrial genomes from extinct and extant rhinoceroses reveals lack of phylogenetic resolution
Source: BMC Evol Biol. 2009 May 11;9:95. doi: 10.1186/1471-2148-9-95 (PMC2694787; doi:10.1186/1471-2148-9-95)
Supplement: Additional file 1 — Figure S1. Nonsynonymous sites in mitochondrial cytb sequences of rhinoceroses, mapped onto the bovine structure [PDF:1PPJ] [22]. The nonsynonymous sites are shown in white, with those located in functionally relevant areas represented as orange spheres, and surrounded by boxes in the alignment. The prosthetic groups are represented in black, and bound inhibitors in brown (ant: antimycin; stig: stigmatellin). Sites that, when mutated in humans, are responsible for exercise intolerance, are depicted in red. [file 1471-2148-9-95-S1.pdf]

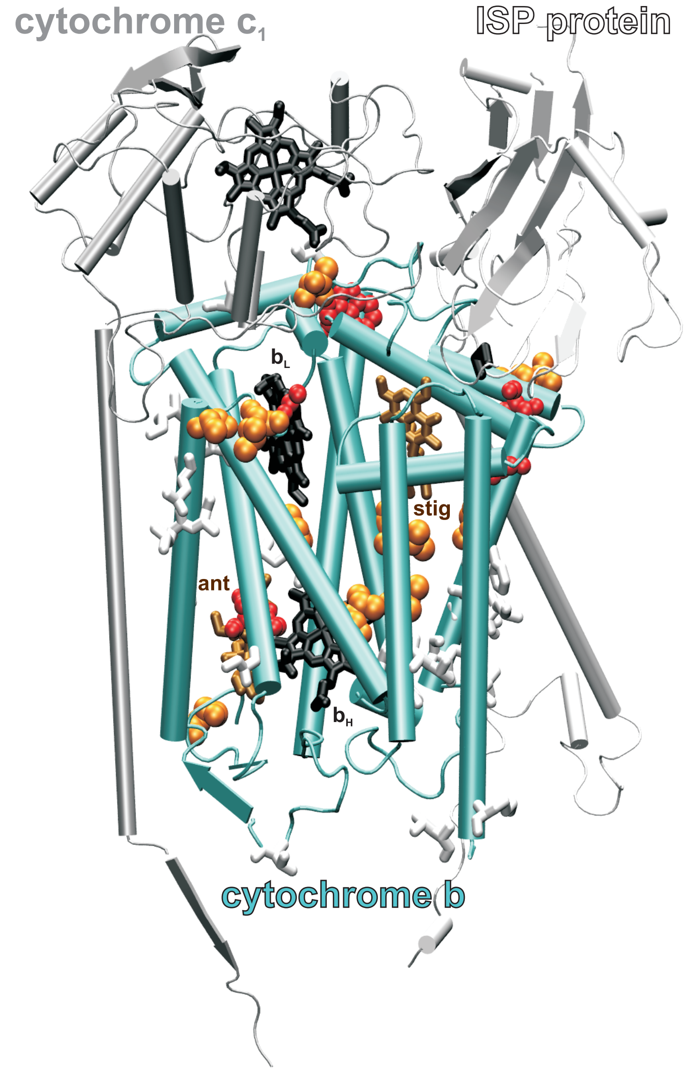

	Javan	Indian	Sumatran	Woolly	White	Black	
	I	V	I	I	I	I	11
	N	N	S	N	N	N	15
	S	S	S	S	A	A	29
	T	T	T	T	M	T	60
cyt c_1	A	T	A	A	A	A	67
cyt c_1	M	M	I	M	I	I	78
cyt c_1	L	L	T	T	L	L	82
b_H	V	V	V	V	V	M	98
	L	L	L	L	I	L	102
	H	Y	Y	Y	Y	Y	107
b_H	I	I	T	T	V	V	117
stig	L	F	L	L	F	F	121
ISP	N	N	D	D	N	N	159
ant	L	L	L	L	M	S	190
cyt c_1	T	M	M	M	M	M	215
	A	A	A	A	I	I	232
cyt c_1	M	L	L	M	L	L	237
cyt c_1	V	V	A	A	A	T	238
ex int	T	I	T	I	A	T	241
ex int	F	F	S	S	S	S	246
cyt c_1	I	I	L	L	I	I	249
stig	I	T	T	T	T	T	257
	T	A	A	T	V	A	295
	F	F	F	F	L	L	296
	I	I	I	I	T	I	300
	L	L	I	I	I	I	303
	I	I	V	I	I	V	304
	S	Y	S	S	F	F	306
	M	M	V	M	M	M	324
	S	S	S	T	T	S	360
	S	N	N	S	N	N	375
	L	L	L	M	L	L	377
