# Supplementary material for: Analysis of complete mitochondrial genomes from extinct and extant rhinoceroses reveals lack of phylogenetic resolution
Source: BMC Evol Biol. 2009 May 11;9:95. doi: 10.1186/1471-2148-9-95 (PMC2694787; doi:10.1186/1471-2148-9-95)

COI

	3	57	106	136	143	144	155	295	404	415	459	469	472	489	490
Black	I	I	P	L	V	D	V	V	T	M	F	V	I	T	S
White	V	V	P	L	V	D	V	V	T	T	F	V	I	T	S
Woolly	I	V	P	M	V	D	V	V	T	T	F	V	V	T	S
Sumatran	I	V	P	M	V	D	V	V	M	T	F	V	V	T	S
Indian	I	V	P	L	V	D	I	V	T	T	L	V	V	S	F
Javan	I	I	S	L	I	N	V	I	S	T	F	M	V	S	S

COII

	5	89	97	126	148	150	165	185	224	227
Black	L	E	V	S	M	I	I	V	A	I
White	F	E	V	S	M	I	V	V	A	L
Woolly	F	E	V	S	M	V	V	V	A	L
Sumatran	F	E	V	P	V	I	V	V	A	L
Indian	L	E	I	S	M	I	V	I	T	L
Javan	L	K	I	S	M	V	V	I	A	L

COIII

	32	40	41	48	51	61	62	73	74	122	136	171	192	213
Black	A	T	L	T	L	I	V	L	V	Y	I	I	I	A
White	A	M	L	M	L	I	V	L	V	H	V	V	I	T
Woolly	V	A	L	T	M	V	I	P	A	H	V	I	I	T
Sumatran	V	M	L	T	M	V	I	P	A	H	V	V	V	T
Indian	V	T	L	T	L	I	I	P	A	H	V	I	V	T
Javan	V	T	F	T	L	I	I	S	A	H	V	I	V	T

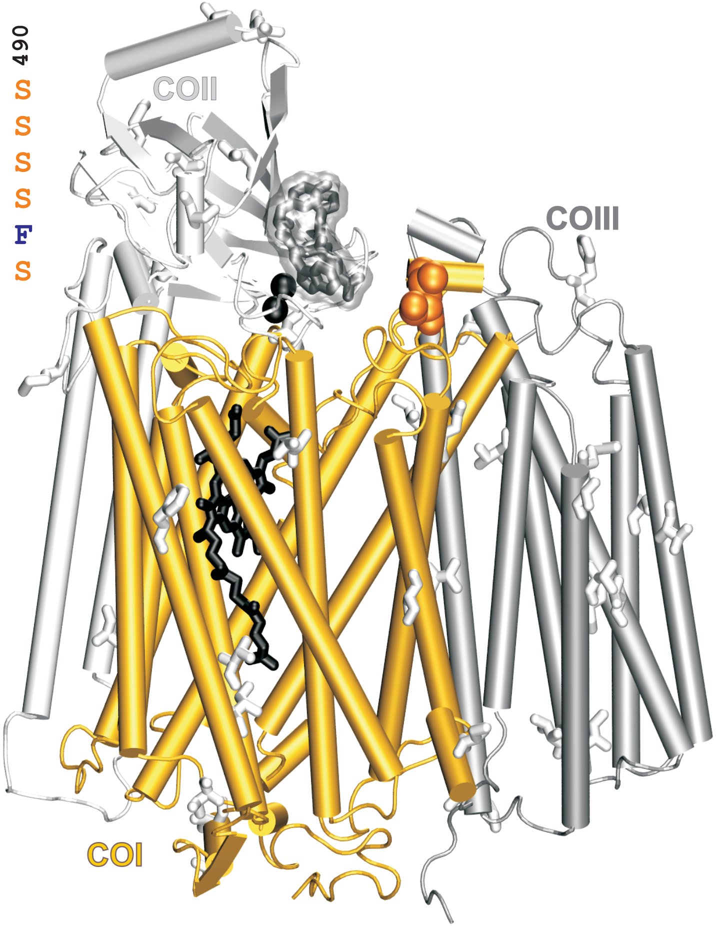

Supplement: Additional file 2 — Figure S2. Nonsynonymous sites in mitochondrial co1/co2/co3 sequences of rhinoceroses, mapped onto the bovine structure [PDF:1V54] [23]. The nonsynonymous sites are shown in white, with that located in a functionally relevant area represented as orange spheres, and surrounded by a box in the alignment. Prosthetic groups are represented as grey spheres. [file 1471-2148-9-95-S2.pdf]
