# Supplementary material for: Analysis of complete mitochondrial genomes from extinct and extant rhinoceroses reveals lack of phylogenetic resolution
Source: BMC Evol Biol. 2009 May 11;9:95. doi: 10.1186/1471-2148-9-95 (PMC2694787; doi:10.1186/1471-2148-9-95)
Supplement: Additional file 3 — Table S1. Details of the primers used for gapfilling of the Javan rhinoceros mitochondrial genome. [file 1471-2148-9-95-S3.doc]

**Table S1.** Details of the primers used for gapfilling of the Javan rhinoceros mitochondrial genome.

| **Forward primer** | **Sequence 5' - 3'** | **Reverse primer** | Sequence 5' - 3' | **Annealing temperature (°C)** | **Size** |
| --- | --- | --- | --- | --- | --- |
| Jav1F | AGCAGGTATCAAGCGCACTA | Jav1R | CCCAGTTTGGGTCTTAGCTG | 58 | 320 |
| Jav2F | CCAATTACCACTAAGCCCACTT | Jav2R | TGGACAACCAGCTATCACCA | 60 | 350 |
| Jav3Fb | GCCATCAATTAAGAAAGCGTCT | Jav3Rb | GGTTTGTTGTTGGATTGTGCT | 60 | 218 |
| Jav4F | GCCACACGAGGGTTTTACTG | Jav4R | GGATTGCGCTGTTATCCCTA | 60 | 317 |
| Jav5Fb | CCCGGTAACTGCATAAAACTT | Jav5Rb | TGGTTGTAGGGGTTCTTTGG | 60 | 216 |
| Jav6F | AAACTAACCGAGCCCCATTT | Jav6R | AAGGCTAGGGTGAGGGGTAA | 60 | 304 |
| Jav7Fb | CATAGCATTCCCCCGAATAA | Jav7Rb | GGCCGGTGGTTTTATATTGA | 60 | 212 |
| Jav8F | CGGAACAGGATGGACTGTCT | Jav8R | TATCCCAAAGCCTGGTAGGA | 60 | 353 |
| Jav9F | GATGCCCTCCTCCGTATCAT | Jav9Rb | GATGGGGGATGTTGCATCT | 60 | 191 |
| Jav9Fb | AAACCATCTGAACGATTCTACCA | Jav9R | TGTGGGGATTATGTAGGAGTCA | 60 | 190 |
| Jav10Fb | GCATCATATTCCCCTCATCC | Jav10Rb | CCATGCCCAGGTTTATTGAC | 60 | 219 |
| Jav11F | CCACAGGCTTTCACGGACTA | Jav11R | GAGAAAGGTAGGCGTGCTGA | 60 | 373 |
| Jav12Fv2 | TCAACACAACAACCTACAGCCTA | Jav12Rv2 | TGGTGCTGGCTGGCTATAAT | 60 | 175 |
| Jav13F | CTCCACCTCCTACCCCTTCT | Jav13Rb | ACGGATAGCTCCCATCCTTT | 60 | 219 |
| Jav14Fb | TGCCAGTAGCCCTCTTTGTT | Jav14Rb | CTTGTAGGGCGGCTGTGT | 60 | 208 |
| Jav15F | CTATCGGCATCAACCAACCT | Jav15R | TTAGTAGGGCTCAGGCGTTG | 60 | 305 |
