# Supplementary material for: Analysis of complete mitochondrial genomes from extinct and extant rhinoceroses reveals lack of phylogenetic resolution
Source: BMC Evol Biol. 2009 May 11;9:95. doi: 10.1186/1471-2148-9-95 (PMC2694787; doi:10.1186/1471-2148-9-95)
Supplement: Additional file 4 — Table S2. Matrix of uncorrected p-distances for the whole mitochondrial genomes of six rhinoceros species, with distances between sister species given in bold. [file 1471-2148-9-95-S4.doc]

**Table S2**. Matrix of uncorrected p-distances for the whole mitochondrial genomes of six rhinoceros species, with distances between sister species given in bold

|  | 1 | 2 | 3 | 4 | 5 |
| --- | --- | --- | --- | --- | --- |
| 1 *Diceros bicornis* | - |  |  |  |  |
| 2 *Ceratotherium simum* | **0.06968** |  |  |  |  |
| 3 *Rhinoceros unicornis* | 0.11218 | 0.10970 |  |  |  |
| 4 *Rhinoceros sondaicus* | 0.11470 | 0.11382 | **0.06181** |  |  |
| 5 *Coelodonta antiquitatis* | 0.11209 | 0.11345 | 0.11300 | 0.11435 |  |
| 6 *Dicerorhinus sumatrensis* | 0.11411 | 0.11250 | 0.11418 | 0.11396 | **0.08638** |
