# Supplementary material for: Analysis of complete mitochondrial genomes from extinct and extant rhinoceroses reveals lack of phylogenetic resolution
Source: BMC Evol Biol. 2009 May 11;9:95. doi: 10.1186/1471-2148-9-95 (PMC2694787; doi:10.1186/1471-2148-9-95)
Supplement: Additional file 5 — Table S3. Details of the components of the aligned mitochondrial genomes from six rhinoceroses, tapir, and horse. [file 1471-2148-9-95-S5.doc]

**Table S3.** Details of the components of the aligned mitochondrial genomes from six rhinoceroses, tapir, and horse

| Component | **Aligned sites** | **Variable sites** | **Parsimony- informative sites** | **Best model**^a^ |
| --- | --- | --- | --- | --- |
| *12S* rRNA loops | 532 | 147 | 76 | TrN+G |
| *16S* rRNA loops | 978 | 291 | 152 | TrN+G |
| D-loop | 798 | 285 | 135 | HKY+G |
| *atp6* | 681 | 240 | 145 | HKY+I |
| *atp8* | 204 | 84 | 43 | TrN+G |
| *cox1* | 1,545 | 487 | 298 | HKY+I |
| *cox2* | 684 | 214 | 123 | HKY+G |
| *cox3* | 783 | 243 | 148 | HKY+I |
| *Cytb* | 1,140 | 372 | 220 | TIM+I |
| *nd1* | 957 | 309 | 150 | HKY+I |
| *nd2* | 1,044 | 361 | 181 | HKY+I |
| *nd3* | 345 | 123 | 67 | HKY+G |
| *nd4* | 1,377 | 503 | 289 | HKY+I+G |
| *nd4l* | 297 | 107 | 59 | HKY+G |
| *nd5* | 1,821 | 659 | 362 | HKY+I |
| *nd6* | 528 | 205 | 112 | HKY+G |
| 1st codon sites | 3,626 | 732 | 372 | GTR+G |
| 2nd codon sites | 3,626 | 281 | 135 | HKY+I |
| 3rd codon sites | 3,626 | 2689 | 1578 | TIM+I+G |

^a^Best model selected by comparison of Bayesian information criterion scores.
