# Supplementary material for: Analysis of complete mitochondrial genomes from extinct and extant rhinoceroses reveals lack of phylogenetic resolution
Source: BMC Evol Biol. 2009 May 11;9:95. doi: 10.1186/1471-2148-9-95 (PMC2694787; doi:10.1186/1471-2148-9-95)
Supplement: Additional file 6 — Table S4. Support for three candidate topologies estimated using Bayesian and maximum-likelihood phylogenetic analysis, using nine laurasiatherian outgroup species. In addition to the six rhinoceros sequences, the following outgroup taxa were included: Tapirus terrestris [GenBank:AJ428947], Equus caballus [GenBank:X79547], Hippopotamus amphibius [GenBank:NC_000889], Equus asinus [GenBank:NC_001788], Artibeus jamaicensis [GenBank:NC_002009], Ursus arctos [GenBank:NC_003427], Manis tetradactyla [GenBank:NC_004027], Bos taurus [GenBank:NC_006853], and Sorex unguiculatus [GenBank:NC_005435]. Phylogenetic analyses were performed on first and second codon positions only, using an unpartitioned GTR+I+G substitution model. Other settings in the Bayesian and likelihood-based analyses were as described in the main text, the only exceptions being the use of the heuristic tree-bisection-reconnection instead of a branch-and-bound search in the maximum-likelihood analysis, and the calculation of the bootstrap support values from 200 pseudoreplicates rather than 1,000. [file 1471-2148-9-95-S6.doc]

**Table S4.** Support for three candidate topologies estimated using Bayesian and maximum-likelihood phylogenetic analysis, using nine laurasiatherian outgroup species. In addition to the six rhinoceros sequences, the following outgroup taxa were included: *Tapirus terrestris* [GenBank:AJ428947], *Equus caballus* [GenBank:X79547], *Hippopotamus amphibius* [GenBank:NC_000889], *Equus asinus* [GenBank:NC_001788], *Artibeus jamaicensis* [GenBank:NC_002009], *Ursus arctos* [GenBank:NC_003427], *Manis tetradactyla* [GenBank:NC_004027], *Bos taurus* [GenBank:NC_006853], and *Sorex unguiculatus* [GenBank:NC_005435]. Phylogenetic analyses were performed on first and second codon positions only, using an unpartitioned GTR+I+G substitution model. Other settings in the Bayesian and likelihood-based analyses were as described in the main text, the only exceptions being the use of the heuristic tree-bisection-reconnection instead of a branch-and-bound search in the maximum-likelihood analysis, and the calculation of the bootstrap support values from 200 pseudoreplicates rather than 1,000.

| **Bayesian  posterior probability** | | | **Maximum-likelihood  bootstrap support** | | |
| --- | --- | --- | --- | --- | --- |
| 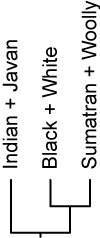 | 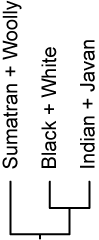 | 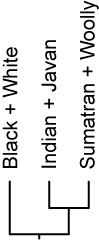 | 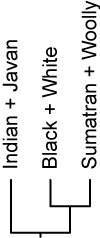 | 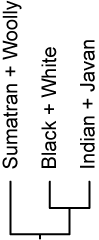 | 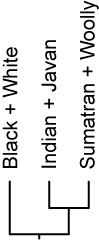 |
| 0.000 | 0.965 | 0.035 | 0.000 | 0.405 | 0.595 |
